# Integrated image and sensor-based food intake detection in free-living

**DOI:** 10.1038/s41598-024-51687-3

**Published:** 2024-01-18

**Authors:** Tonmoy Ghosh, Yue Han, Viprav Raju, Delwar Hossain, Megan A. McCrory, Janine Higgins, Carol Boushey, Edward J. Delp, Edward Sazonov

**Affiliations:** 1 Electrical and Computer Engineering Department, University of Alabama, Tuscaloosa, AL 35401 USA; 2 Electrical and Computer Engineering Department, Purdue University, West Lafayette, IN 47907 USA; 3 Department of Health Sciences, Boston University, Boston, MA 02215 USA; 4 Department of Pediatrics-Endocrinology, University of Colorado, Denver, CO 80045 USA; 5 Epidemiology Program, University of Hawaii Cancer Center, Honolulu, HI 96813 USA

**Keywords:** Computational models, Data mining, Data processing, Image processing, Biomedical engineering

## Abstract

The first step in any dietary monitoring system is the automatic detection of eating episodes. To detect eating episodes, either sensor data or images can be used, and either method can result in false-positive detection. This study aims to reduce the number of false positives in the detection of eating episodes by a wearable sensor, Automatic Ingestion Monitor v2 (AIM-2). Thirty participants wore the AIM-2 for two days each (pseudo-free-living and free-living). The eating episodes were detected by three methods: (1) recognition of solid foods and beverages in images captured by AIM-2; (2) recognition of chewing from the AIM-2 accelerometer sensor; and (3) hierarchical classification to combine confidence scores from image and accelerometer classifiers. The integration of image- and sensor-based methods achieved 94.59% sensitivity, 70.47% precision, and 80.77% F1-score in the free-living environment, which is significantly better than either of the original methods (8% higher sensitivity). The proposed method successfully reduces the number of false positives in the detection of eating episodes.

## Introduction

Dietary monitoring is critical for understanding body weight dynamics in humans associated with underweight, overweight, and obesity^1^. Underweight people are more likely to have malnutrition, heart irregularities, bone fractures, and even death^2^. Being overweight increases health risks, for example, type-2 diabetes, cardiovascular diseases, and asthma caused 3.4 million deaths in 2016^3^. Eating behaviors associated with being underweight, or overweight, can be understood by monitoring dietary patterns. Traditional methods for monitoring food intake are food records, 24 h recalls, and food frequency questionnaires^4^. These methods are usually inaccurate and suffer from misreporting^5^ as well as placing an extra burden on the user.

Numerous technology-driven dietary monitoring methods have been suggested. These include manual image capture and various types of wearable sensors. Image-based methods are classified into two types. (1) Active image capture, where the user captures images (e.g. on a smartphone), and (2) Passive image capture, where images are automatically captured without user participation (e.g., by a wearable camera). The advantage of using images is that they capture the foods being eaten. In studies of^6–8^, and^9^, a smartphone-based active image-capturing method was proposed for dietary assessment. Active user involvement is required for smartphone-based approaches, thus placing the burden on the user and increasing the risk of missing images. In^10,11^, and^12^, wearable cameras were used for the dietary assessment was proposed. Other than putting on the device, the passive image capture does not require any human intervention. However, a wearable camera creates privacy concerns as the user is not in control of the image capture^13^. In the study of^9^, food, and drink images were recognized using a deep neural network, the authors called their network ‘NutriNet’, which is a modified version of the popular AlexNet network^14^. This method classified food and drink images from non-food images, however, did not detect eating episodes. Similarly, in^15^, a convolution neural network (CNN) was introduced to recognize food images. To identify food items, image segmentation, and object classification methods were proposed in^16^. The authors also integrated contextual information (user’s diet) to correct potential misclassification. Another deep learning-based method was proposed in^12^, the authors used a wearable camera to capture egocentric images and then classified food and non-food images in real-life scenarios. The food intake detection accuracy (86.4%) was satisfactory. However, this method results in a high number of false positives (13%). In free-living conditions, image-based dietary assessment suffers from false positive detection due to a wearable camera capturing food images that were not consumed by the user.

Sensor-based methods have their advantages and limitations. Various wearable sensors have been proposed to detect eating proxies such as chewing, swallowing, jaw movements, and hand-to-mouth gestures. Acoustic sensors (e.g., microphone) have been used to detect chewing and swallowing sounds^17–23^ and thus detect eating episodes. Another commonly used sensor is the strain sensor that can capture jaw movement^21,24^, throat movement^25–27^, temporal muscle movement^28,29^, and hence detect food intake. Although the food (solid) intake detection accuracy is impressive, strain sensors need direct contact with the skin, which is inconvenient for users. Physiological sensors such as Electromyography (EMG)^30,31^, Respiratory Inductance Plethysmography (RIP)/breathing sensor^32,33^, and glucose monitoring sensor^34–36^ were used to detect eating episodes. The sensors have their advantages and limitations. Motion sensors such as an accelerometer, and gyroscope have also been proposed to detect food intake by hand-to-mouth gesture^37–40^, and head movement^29,41,42^. The main advantage of using motion sensors is that it is convenient to use (no direct contact is necessary), however, they also generate false detection (in the range of 9–30%).

Studies^28,29^ conducted by our research team revolve around the application of chewing sensors, including piezoelectric and flex sensors, to facilitate the detection of food intake. Notably, the identification of eating episodes can be derived from either sensor data or images, given the presence of both chewing and image sensors within the wearable device. Either method may produce false-positive detection. For example, gum chewing may register as an eating episode on chewing sensor data. Foods not being eaten may be recognized in the images from the egocentric camera. Therefore, integrating both sensor-based and image-based food detection methods is essential for attaining more precise and accurate insights into food intake. A separate research^43^ inquiry applied Score-Level and Decision-Level Fusion of Inertial and Video Data for the detection of intake gestures. In this scenario, the inertial sensor is incorporated into wearable devices, while the video camera remains fixed in place. However, implementing such a setup in real-life scenarios, especially free-living situations, presents challenges. In this paper, we bridge this gap and propose a novel food intake detection method that integrates sensor- and image-based detection from wearable sensors.

We use methods from deep learning to recognize solid foods and beverages in images captured by AIM-2. We use sensor-based detection of eating and hierarchical classification to combine confidence scores from both image and sensor methods for accurate eating detection. The paper is organized as follows, first, the Material and Methods are presented in Section “Material and methods” followed by results are discussed in section “Results”. Sections “Discussion” and “Conclusion” are discussion, and conclusion, respectively.

## Material and methods

### Sensor system

**Figure 1:**
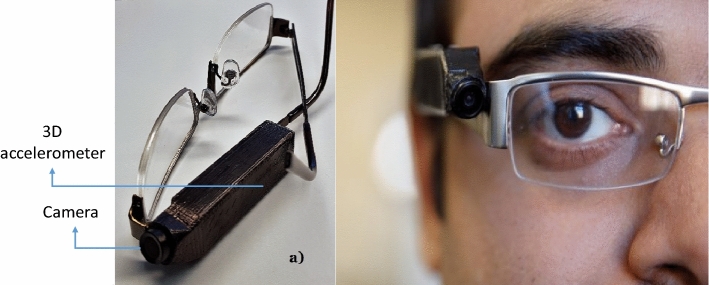
AIM-2 installed on eyeglasses with non-corrective lens.

The Automatic Ingestion Monitor v2, AIM-2^29^ was used in this study. The sensor system was attached to the frame of a pair of glasses. Figure 1 depicts an AIM-2. We used images, captured by the AIM-2 camera and sensor data, collected by the 3D accelerometer (chewing sensor) in this paper. The camera continuously captured images at a rate of one image every 15 s from the user’s egocentric point of view. These images were used to develop image-based food and beverage object detection. The 3D accelerometer sensor recorded head movement as well as head angle and body leaning forward motion, which was used as eating proxies to detect eating episodes. The accelerometer data were sampled at 128 Hz. Data from accelerometer sensors and images were saved to an SD card and processed offline for algorithm development and validation.

### Data collection and ground truth annotation

A study was carried out in order to develop classification methods and assess the accuracy of food intake detection. From August 2018 to February 2019, 30 participants (20 males and 10 females, mean SD age of $23.5 \pm 4.9$ years, range $18 - 39$ years, and mean body mass index (BMI) $23.08 \pm 3.11 \;\;{\text{kg/m}}^{{2}}$) were recruited. The University of Alabama’s Institutional Review Board (IRB) approved the study. The experiment included a pseudo-free-living day and a free-living day. During the pseudo-free-living day, participants consumed three meals in the lab while engaging in otherwise unrestricted daily activities. There were no restrictions on food intake or activities during the free-living day. A detailed description of the study protocol and how the sample size was determined were reported in^29^. All methods were carried out in accordance with the approved IRB’s guidelines and regulations. All subjects have given their informed consent to the use of the recorded data (sensor and image) for presentations, publications, or other forms of dissemination.

During food consumption in the lab, participants used a foot pedal connected to a USB data logger to record food ingestion. They were instructed to press the pedal as soon as they placed the food in their mouth (a bite) and held it until the food was swallowed. Similarly, for liquid food, they were requested to press and hold the pedal from the moment the liquid was placed in their mouth until the last swallow. This foot pedal record was used as ground truth for the pseudo-free-living day to train a food intake detection model for chewing sensor data. The participants continued free living for 24 h after completing the first day (pseudo-free living). The device captured continuous images (one image every 15 s), which were then manually reviewed to extract the ground truth of food intake detection of a free-living day. The number of eating episodes, as well as the start and end times of eating, were annotated and used for validation during the free-living day. During pseudo-free-living days, 372 h of data were collected, capturing 90 meals and a total of 89,257 images (consisting of 3996 food images and 16.65 h of eating). Conversely, in free-living days, 380 h of data were collected, capturing 111 meals and a total of 91,313 images (consisting of 4933 food images and 20.55 h of eating)^29,44,45^. This study exclusively utilizes free-living data due to concerns about potential biases introduced in image-based detection when participants consume their food in a laboratory environment during pseudo-free-living days.

Images from free-living days were annotated by hand with the rectangle bounding box in order to train a classifier to detect food and beverage objects. Initially, all the images were divided into two groups:

(1) Positive samples (contained food/beverage objects) and (2) negative samples (did not contain food/beverage objects). The negative sample images were not annotated and were used directly in the training dataset. Positive images of 30 participants, on the other hand, were annotated, and all food and beverage objects were labeled using MATLAB 2019 Image Labeler application^46^. The annotator did not label food and beverage objects when the scene was food preparation and shopping. Furthermore, annotating during social eating was difficult because food and beverage objects could belong to a different person. As a result, the annotator did not label the food and beverage objects that were far from the subject, assuming that the subject did not consume them. We found a total of 190 food and beverage items.

We used two methods for reporting performance when training, validating, and testing the proposed method: leave one subject out validation and holdout validation. Classifiers were trained and tested using the leave-one-subject-out (LOSO) cross-validation technique to assess performance. This means that data from one participant were kept for testing while data from the other participants were used to train the classifier. It ensured that the classifier never saw the testing data for a specific subject. The procedure was repeated 30 times, so each participant was only tested once. Furthermore, in order to compare the performance of different methods, the dataset was randomly divided into training (80%), validation (10%), and testing (10%) sets for holdout validation which may result same subject data on the training and testing set.

### Image-based food and beverage object detection

**Figure 2:**
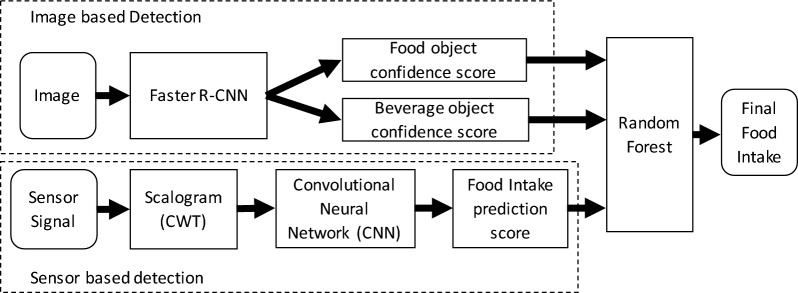
Block diagram of proposed method. Left top block: image based detection, left bottom block sensor based detection and Random forest classifier for integrating both detections.

The proposed method is divided into three parts, (1) image-based detection, (2) sensor-based detection, and (3) Integration of image- and sensor-based prediction. The flow chart of the proposed method is presented in Fig. 2. Recently, CNN-based deep learning methods have shown very good performance in visual recognition. We used Faster R-CNN^47^ to detect food/beverage objects in images captured by AIM. Faster R-CNN is a two-stage detection framework to generate bounding boxes and class labels simultaneously. In order to obtain better recognition results, we adopted transfer learning and used the model pre-trained on ImageNet^48^ as our starting point for training.

**Figure 3:**
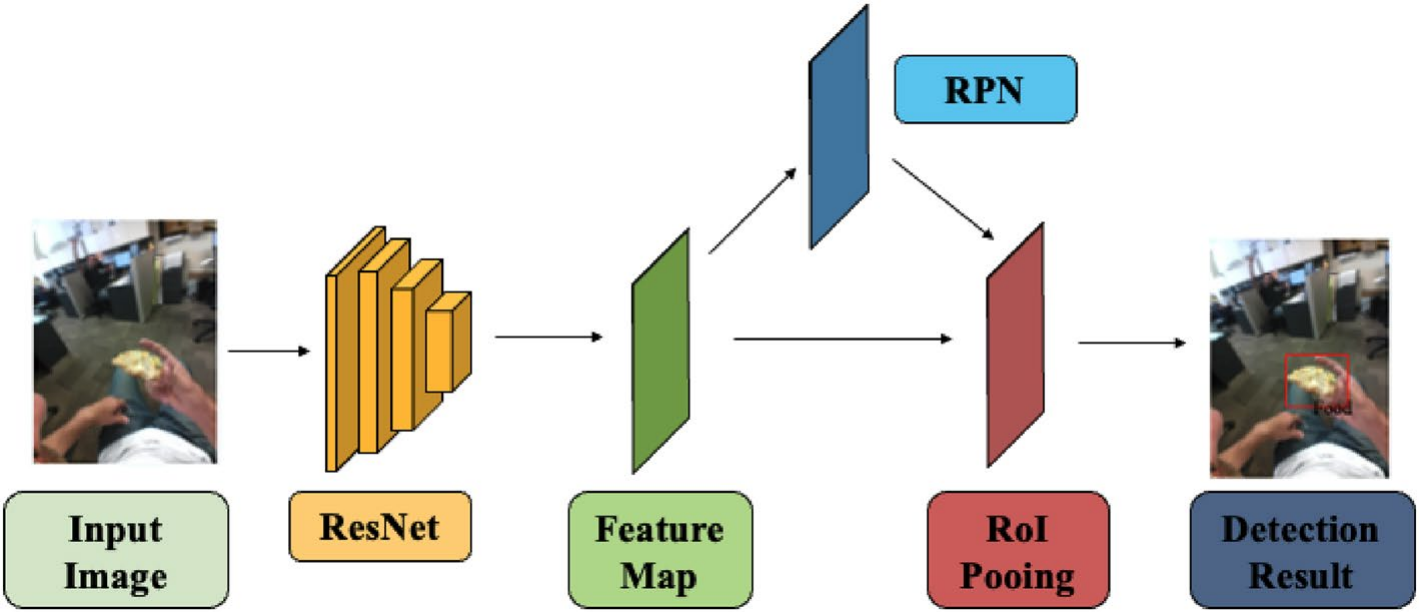
Block diagram of Faster R-CNN based food and beverage object detection. ResNet is used as the feature extractor, RPN generates object proposals, and RoI pooling aligns the extracted feature map for classification and regression.

The block diagram of Faster R-CNN we used is shown in Fig. 3 with example inputs with results. ResNet^48^ is used to extract feature maps from the input image, which are then used by the region proposal network (RPN)^47^ to identify areas of interest in the image from the multi-scale features, 1000 box proposals with confidence scores are obtained in this step. The region of interest (ROI) pooling layers crops and wraps the feature maps using the extracted and generated proposal boxes to obtain fine-tuned box locations and classify the food objects in the image.

In order to obtain better recognition results, we finetuned the Faster R-CNN by using the pre-trained weights of ResNet-50 on ImageNet^48^ as our starting point for training. The Faster R-CNN was trained on training sets for 150 epochs with a batch size of 64 and a learning rate of 2.5e^−4^. The prediction scores obtained from this method were used in the hierarchical classification step.

### Sensor-based food intake detection

The sensor-based food intake detection model was developed using a 3-axis accelerometer sensor signal. The accelerometer sensor recorded head movement, head direction, and temporalis muscle movement, which are used as a proxy for eating detection^41,42^. A Convolutional Neural Network (CNN) replaced the hand-crafted features reported in^41,42^. The signal was first segmented using a 15-s fixed rectangular window (total 15*128 = 1920 samples, where 128 Hz is the sampling frequency), which is called a segment. This window size was selected because it corresponds to the image capture interval. The continuous wavelet transformation (CWT) was then used for each segment. CWT represents the frequency spectrum of a signal as it changes over time. CWT was calculated considering a signal $s\left( \tau \right)$ of length $N$ and the mother wavelet $\psi$:

1 $$C\left( {a,b} \right) = \frac{1}{{\sqrt {\left| a \right|} }}\mathop \smallint \limits_{ - \infty }^{\infty } s\left( \tau \right)\psi \left( {\frac{\tau - b}{a}} \right)d\tau$$

where *a* is the scale and *b* is the translational value. In this analysis, we choose the Morse mother wavelet^49^ and the CWT implemented in MATLAB from MathWorks (e.g. the ‘cwtfilterbank’ function). The scalogram is the absolute magnitude of the CWT, which is calculated as follows:

2 $$scalogram, \;\;S = \left| {C\left( {a,b} \right)} \right|$$

The scalogram was first normalized from 0 to 1 (unit-based normalization). The values are then multiplied by 255 and converted to 8-bit unsigned integer values using the below equation. The conversion process is needed to save the scalogram as an image format.

3 $$I\left( {x,y} \right) = uint8\left( {255*\frac{{S\left( {x,y} \right) - S_{min} }}{{S_{max} - S_{min} }}} \right)$$

**Figure 4:**
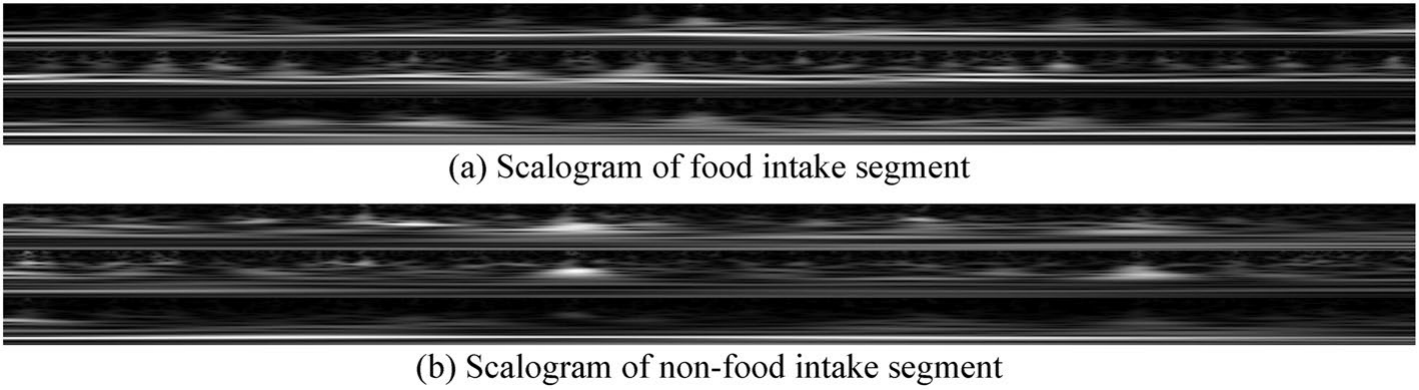
Sample scalogram of food intake and non-food intake segment.

**Figure 5:**
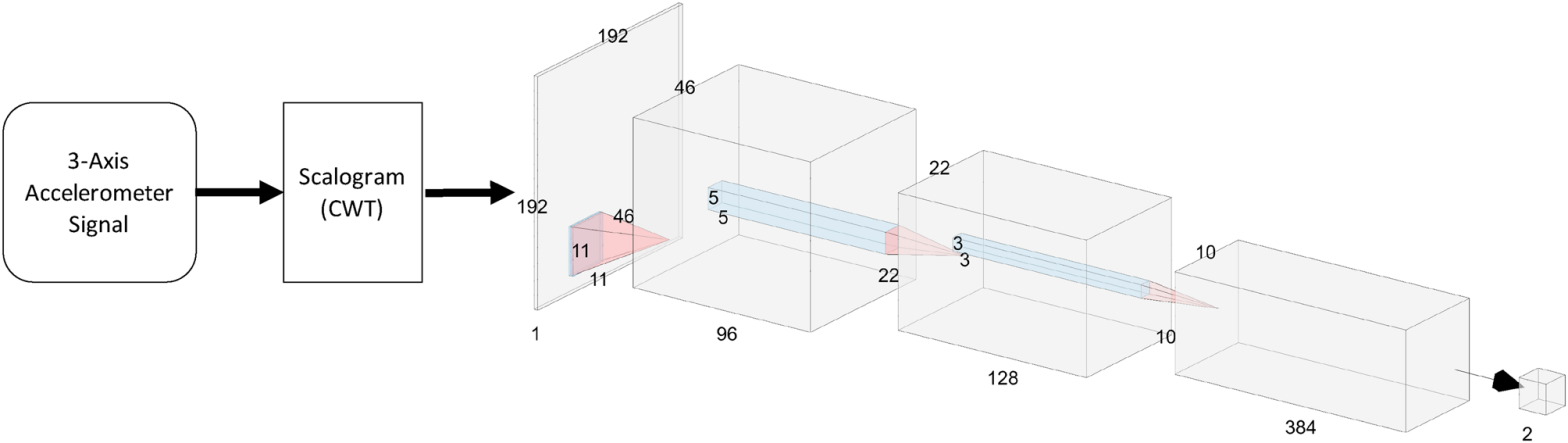
Proposed Convolutional Neural Network Architecture for sensor-based food intake detection.

The size of the scalogram was $64 \times 1920$ pixels (64: frequency components of $1 - 64$ Hz, maximum frequency captured by the accelerometer was 64 Hz due to 128 Hz sampling frequency; 1920: number of samples in a segment). The scalograms obtained from the accelerometer’s three axes were concatenated to produce a final scalogram with a size of $192 \times 1920 \times 1$ ($64 \times 3 = 192$). Eventually, we modified the shape to [192 192 1] by employing under-sampling techniques. Bilinear interpolation served as the method for down-sampling, where the resulting value is computed as a weighted average of data values within the closest 2-by-2 neighborhood. This adjustment resulted in a simplified network structure and a significant reduction in the number of learnable parameters. The transformed data was then saved in the form of an image and called a scalogram. An example of the scalogram of food and non-food intake segment is presented in Fig. 4. A 15-layer CNN architecture was proposed to classify food intake and non-food intake segments. The CNN has three convolutional layers, three ReLu (rectified linear units) layers, three max-pooling layers, two cross-channel normalization layers, one dropout layer, one fully connected layer, one Softmax layer, and finally one classification layer. A convolutional neural network (CNN) employing a 1-dimensional kernel is limited to capturing local dependencies, whereas a CNN utilizing a 2-dimensional kernel has the capability to capture both local and spatial dependencies^50,51^. Since the proposed method used a 2-dimensional kernel, it is extracting features utilizing both the local and spatial dependencies of scalograms. This network is graphically represented in Fig. 5. The network was trained on training sets on 8 epochs with a batch size of 32. The batch size of 32 has been selected primarily because of memory constraints on the trained computer and epoch number is obtained by monitoring the loss function to prevent overfitting.

### Integration of image- and sensor-based prediction

In order to combine the image and sensor methods, we adopted state-of-the-art approaches such as bagging and boosting^52^. The inputs were the confidence scores of the image- and sensor-based detection. Bagging is a voting-based method to combine multiple learners. In bagging, we investigated random forest^53^, which is an ensemble decision tree classifier. Among boosting methods, we tried adaptive boosting^54^, and random under-sampling boosting (RUSBoosted)^55^ classifiers. Linear discriminant, subspace discriminant, logistic regression, gaussian Naïve Bayes, and K-nearest neighbor classifier have also been tested and their performance in detecting eating episodes was evaluated. The best method was selected, which achieved the best performance.

Furthermore, temporal information was considered in order to improve performance even further. To predict food intake detection at the time $t$, prior confidence scores $t - n, \left( {n = 1, 2, \ldots } \right)$ are also used as predictors. Let, the confidence scores of a food object, $S_{f}$, beverage object, $S_{b}$ (if multiple food/beverage objects were detected, the highest confidence score was counted), and sensor prediction, $S_{s}$, then the predictor vector at time $t$ is as follows:

$$\left[ {\begin{array}{*{20}c} {S_{f} \left( t \right) \ldots S_{f} \left( {t - n} \right)} & {S_{b} \left( t \right) \ldots S_{b} \left( {t - n} \right)} & {S_{s} \left( t \right) \ldots S_{s} \left( {t - n} \right)} \\ \end{array} } \right]$$

In this analysis, we considered $n = 0, 1, 2$. It is to be noted that the confidence scores were acquired on a segment basis, such as within a 15-s interval. Thus, when $n = 0$, the system evaluated a 15-s signal alongside a single image. Subsequently, for $n = 1$, it analyzed a 30-s signal ($15*\left( {n + 1} \right)$) along with two images, and this pattern continued for subsequent values of n. The optimal value of n was determined by analyzing the food intake performance in a grid search approach. The process of Grid search encompassed the range of values for n, starting from $n = 0$ and progressing to $n = 4$, with an increment of 1.

### Performance criteria

To validate the performance, four commonly used performance criteria were computed: sensitivity, specificity, F1-score, and accuracy^56^. These are defined as:

4 $$Sensitivity = \frac{TP}{{TP + FN}}$$

5 $$Precision = \frac{TP}{{TP + FP}}$$

6 $$Accuracy = \frac{TP + TN}{{TP + FP + TN + FN}}$$

7 $$F1 score = \frac{2TP}{{2TP + FN + FP}}$$

where TP denotes a true positive, a ‘food intake’ event correctly detected as ‘food intake’; TN denotes a true negative, a ‘non-food intake’ event correctly detected as ‘non-food intake’; FN denotes a false negative, a ‘food intake’ event incorrectly detected as ‘non-food intake’; and FP denotes a false positive, a ‘non-food intake’ event. It should be noted that for this binary classification, all eating activities, including drinking beverages, are collectively categorized as ‘food intake’. Furthermore, we also reported performance results in terms of eating episode detection. During a standard eating episode, a bite is succeeded by a series of chews and swallows, and this cycle is reiterated until a portion of food is consumed to fulfill one's appetite^29^. Moreover, eating events from an individual with a < 15 min inter-event interval were combined into one eating episode^57^.

We used mean Average Precision (mAP) as our evaluation metric for image-based food and beverage detection. The predicted detection is considered a true positive (TP) if the detected label equals the ground-truth label, and the overlap ratio of the IoU (Intersection over Union) between the detected bounding box and ground truth is not smaller than a predefined threshold. The Average Precision (AP) is calculated as the area under the Precision-Recall curve, which involves computing Precision and Recall at various confidence score thresholds ranging from 0 to 1 with an increment of 0.1. The mean Average Precision (mAP) is then obtained by averaging the AP scores over a set of IoU thresholds. We choose to use the mAP with a set of IoU thresholds from 0.5 to 0.95 with a 0.05 increment, which is a generalized metric and serves as the main evaluation metric for the MS COCO object detection challenge^52^. We denote this metric as mAP@[.5,.95], which serves as a strict criterion for evaluating object detection methods. For reference, the current Top-1 result in the MS COCO object detection challenge achieves an mAP@[.5,.95] of 58.8.

Furthermore, we used McNemar’s test^52^ to determine if the performances of the two classification methods were statistically similar or different. Let, $e_{01} :$ the number of eating episodes misclassified by Classifier-1 but not Classifier-2, and $e_{10} :$ the number of eating episodes misclassified by Classifier-2 but not Classifier-1. The classification methods have the same error rate under the null hypothesis, we expect $e_{01} = e_{10}$ and these equal to $(e_{01} = e_{10} )/2$. The chi-square statistic of one degree of freedom was calculated as follows.

8 $$\frac{{\left( {\left| {e_{01} - e_{10} } \right| - 1} \right)^{2} }}{{e_{01} + e_{10} }}\sim \chi_{1}^{2}$$

McNemar’s test rejects the hypothesis that the error rates of the two classification methods are the same at the significance level of α if this value is greater than $\chi_{\alpha ,1}^{2}$. For $\alpha = 0.01, \chi_{0.01,1}^{2} = 6.635$.

## Results

**Figure 6:**
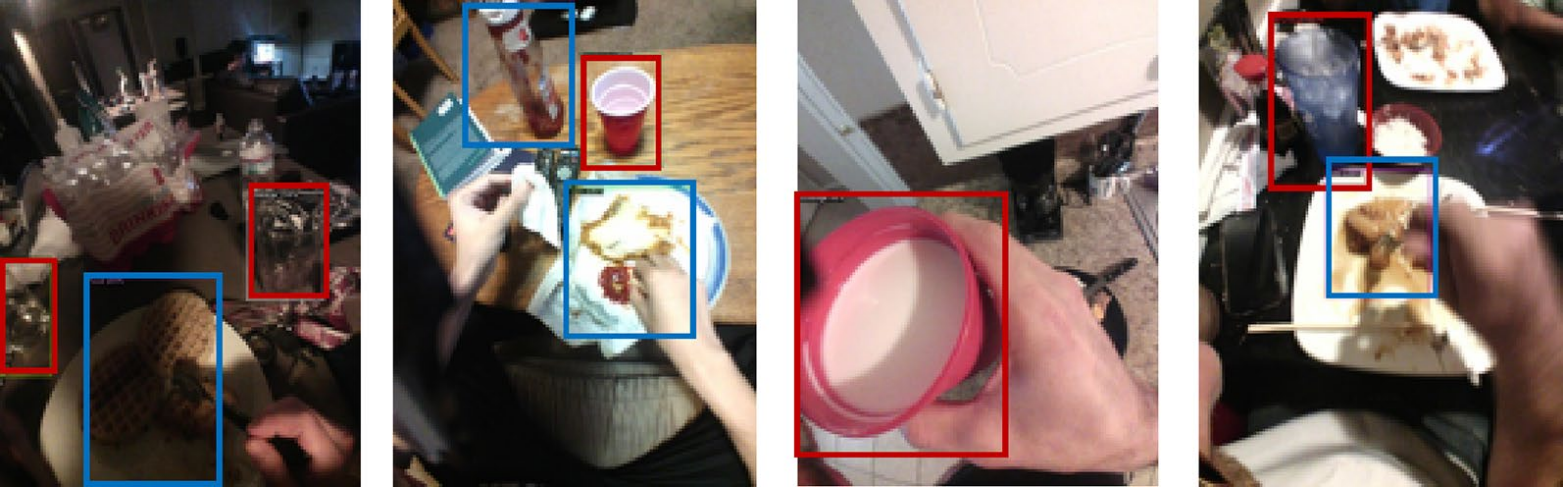
Example of food and beverage recognition (blue bounding box—solid food, red—beverages).

Table 1 shows the results of image-based food and beverage detection. The overall detection performance achieved using the holdout validation technique is 51.97 mAP@[.5,.95] score, which is an excellent result considering the variety of foods and beverages (190 items). In LOSO cross-validation, the detection performance dropped to 20.10 mAP@[.5,.95]. Because the food items of each subject differed, the trained classifier was unable to detect food objects that it had not previously seen. Figure 6. shows examples of food and beverage detection. It demonstrates that the proposed algorithm successfully detected both food and beverage objects. It is important to note that all the results presented in this paper are derived solely from data collected during free-living days.

**Table 1:** Performance (mAP@[.5,.95]) of food and beverage object detection.

Object	Holdout validation	LOSO cross validation
Food	50.13	19.94
Beverage	53.81	20.25
Overall	51.97	20.10

Performance results of sensor-based food intake detection on LOSO cross-validation are presented in Table 2. The performance results are reported in terms of the mean and standard deviation of each subject’s performance. 77.55% F1-score was obtained using the proposed CNN architecture. The performance outcomes were based on 15-s segment-based detection. It also produced a good number of false-positive detections.

**Table 2:** Food and non-food intake classification performance using only sensor-based classifier.

	Sensitivity (%)	Precision (%)	Accuracy (%)	F1-score (%)
Mean	73.21	84.48	86.93	77.55
Standard deviation	12.21	8.92	5.49	7.94

Table 3 shows the performance of the integrated classifier. We used the holdout validation technique to find the best classifier, and the performance results are reported on a segment basis. The best precision and F1-score were obtained using the Random Forest classifier (parameters: Maximal number of decision splits (or branch nodes) per tree = 1000, Number of ensembles learning cycles = 30, Misclassification cost = [0 1; 2 0] two times for food intake segment). It provided the best performance because this problem is non-linear, and the ensemble approach assisted in overcoming critical cases. Thus, in this proposed method, we choose a random forest classifier to integrate image and sensor detection.

**Table 3:** Performance comparison among integrated classifiers.

Method	Sensitivity (%)	Precision (%)	Accuracy (%)	F1 (%)
Adaptive boosting	77.68	90.54	98.66	83.62
Random forest	90.61	90.55	99.17	90.58
LDA	77.11	75.50	97.89	76.30
RUSBoosted	95.10	55.40	96.42	70.01
Subspace Discriminant	77.21	75.56	97.90	76.37
Logistic Regression	69.78	73.68	97.58	71.68
Gaussian Naïve Bayes	94.05	42.52	94.15	58.57%
KNN	86.58	93.23	99.13	89.78

Moreover, classification performance using temporal confidence scores is presented in Table 4. In Table 4, the random forest classifier was used. It is observed that after adding the confidence scores from previous segments/images, the classification performance improved. For example, in the case of $n = 1$, sensitivity and F1-score were improved by 1% and 2%, respectively. Likewise, increasing the value of $n$ progressively enhances performance. A higher $n$ value yields improved results in food intake detection; nevertheless, it necessitates reliance on larger sets of preceding confidence scores. Moreover, increasing $n$ may lead to better performance in large meals but miss small eating episodes. The dataset is too small to explore this thoroughly.

**Table 4:** Analysis of food intake detection performance using temporal confidence scores.

n	Sensitivity (%)	Precision (%)	Accuracy (%)	F1-score (%)
0	90.61	90.55	99.17	90.58
1	91.58	93.84	99.37	92.70
2	93.06	95.33	99.49	94.18
3	93.81	96.25	99.57	95.01
4	94.49	96.79	99.62	95.63

n is the number of previous confidence scores added to the predictor vector.

Finally, the performance of the integrated classifier was compared using the LOSO cross-validation technique, as shown in Table 5. To enable a meaningful comparison with the state-of-the-art method^29^, here, performance is measured in terms of eating episodes. In terms of sensitivity, the integrated classifier outperformed the sensor- and image-based methods by 8% and 6%, respectively. In comparison, the integrated method improved the precision and F1-score by up to 20% and 17%, respectively, over the image-based method. The integrated classifier was chosen due to its capacity to improve sensitivity while preserving the f1 score at a minimal reduction of just 0.24% when compared to the sensor-based method.

**Table 5:** Performance matrices for free-living experiments.

Method	Sensitivity	Precision (%)	F1 (%)
Image	88.29%	50.00	63.64
Sensor	86.49%	76.19	81.01
Integrated (image and sensor)	94.59%	70.47	80.77
Doulah et al.^29^	57.83% (*72.73%)	–	–

*Only solid food (not including beverage).

McNemar’s test was used to determine whether the performance of the Image, Sensor, and Integrated classification methods was statistically similar or different. The number of misclassified eating episode detection metrics was used in this test. We started by comparing the image-based classifier (C1) and the integrated classifier (C3). Due to a high number of false eating episodes detected by C1, the number of eating episodes misclassified by C1 but not C3 was $70$. And the number of eating episodes misclassified by C3 but not C1 was 6. Thus, the calculated chi-square was $\chi_{1}^{2} = 52.22$. McNemar’s test rejected the hypothesis at a significant level $\alpha = 1\%$, due to the $\chi_{1}^{2}$ is greater than 6.635. Similarly, in the same way, we also tested sensor-based classifier (C2) with integrated classifier (C3) and found $\chi_{1}^{2} = 7.09$. Thus, McNemar’s test also rejected the null hypothesis that the two classification methods have the same error rate at a significant rate $= 1\%$. So, the integrated classification method is statistically different than image-based and sensor-based methods.

## Discussion

The primary goal of this research was to develop and validate an accurate food intake detection method. Following the main goal, this work first demonstrated the method for detecting food and beverage objects from images, then food intake detection using sensor signals, and finally combining those detections to obtain final food intake detection. The overall AP score for food and beverage object detection is 51.97, which is a good object detection score. However, this object detection cannot tell whether the detected food was consumed by the user. For example, if a person is preparing food, cooking food, or socializing, the food object in front of that person can be detected, resulting in false-positive food intake detection. The accelerometer-based food intake detection algorithm, on the other hand, is 86.59% accurate for ingestion events.

The main disadvantage of the sensor-based method is that it cannot detect drinking episodes because there is no chewing involved. However, it may detect false positives in the case of chewing gum. The image-based method, on the other hand, detects false eating episodes because it cannot distinguish whether a food is consumed or not. In this paper, we combined image and sensor-based detection to address these shortcomings. Because it successfully removed false detection of individual sensors, we saw a significant improvement in food intake detection performance. The integrated technique finds more actual eating episodes (due to increased sensitivity). When compared to image-based food and beverage detection, it improves detection on all performance criteria, including sensitivity, precision, and f1-score.

**Figure 7:**
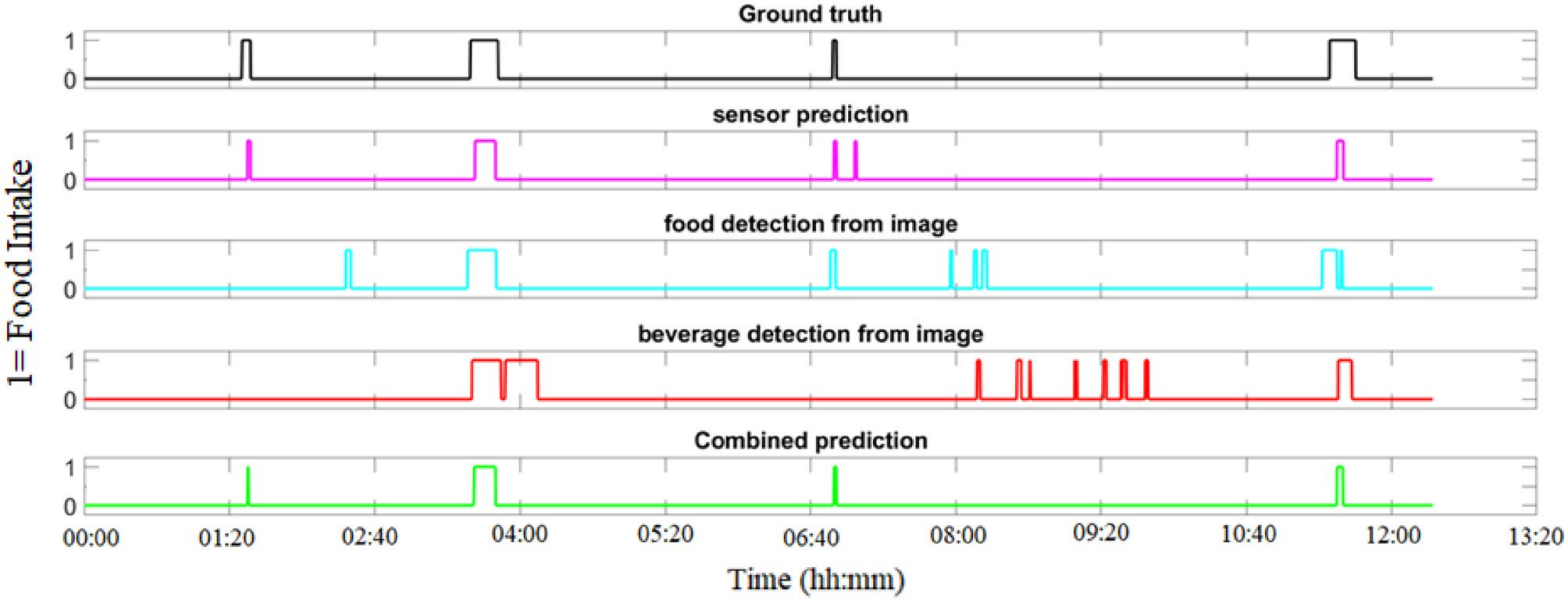
Food intake detection outcome obtained using proposed integrated method. Top to bottom: ground truth, sensor-based classifier, image-based food detection, image based beverage detection and Integrated (image and sensor) based classifier.

Additionally, we compared the proposed method to a recently published method^29^. When considering both solid foods and beverages, a significant performance improvement (37% more sensitive) was achieved. One of the most significant contributions of this paper is its ability to detect both solid and liquid dietary intake. This performance improvement was achieved because the proposed method successfully eliminated false-positive detection and can detect both solid food and beverage intake. Figure 7 shows a demonstration of food intake detection using an integrated two-stage classifier. Note that for this subject, false positives (eating detections that do not match eating episodes in the ground truth data) have been eliminated.

We observed a decrease in performance on food and beverage object detection using the LOSO cross-validation technique. In order to improve object detection performance, we will train the classifier with more image data that includes a wider range of background scenes and food items in the future. Furthermore, we investigate sensor-based detection failure cases. We discovered that it failed in the case of short-eating events or snacks, such as a bite of chips, a few bites of semi-solid yogurt, and a small chocolate. Those eating episodes are extremely difficult for any sensor to detect (chewing sensor or image). The integrated method fails to detect those as well. An additional constraint of this proposed method is the relatively small image dataset, consisting of only 190 types of foods and beverages. To cultivate a resilient food and beverage detection algorithm, a more substantial volume of data is requisite for training. Moreover, most people (in free-living) eat their meals socially, thus it is very difficult to label food/beverage items of that social eating.

## Conclusion

Automatic food intake detection in a free-living environment is a difficult task. This study showed that integrating image and chewing sensor (accelerometer) based prediction provides accurate and precise performance. In terms of eating episode detection, the proposed method achieved 94.59% sensitivity, 70.47% precision, and 80.77% f1-scores. It can detect both solid and liquid dietary consumption. Accurate detection of eating episodes in free-living may benefit from incorporating multiple sources into the decision-making process. In future developments, the proposed approach holds potential for deployment on a cloud-based server, enabling the provision of remote monitoring data. Moreover, there is scope to extend the method to encompass other food intake monitoring metrics, such as chewing/eating rate, dining environment analysis, and estimation of total caloric intake.

## Data Availability

The data used in this paper are protected under the University of Alabama IRB. The dataset may be made available upon request contingent on establishing an inter-institutional data sharing agreement.
